# Configurations of Proto-Cell Aggregates with Anisotropy: Gravity Promotes Complexity in Theoretical Biology

**DOI:** 10.3390/e24111598

**Published:** 2022-11-03

**Authors:** Juan César Flores

**Affiliations:** Departamento de Física, FACI, Universidad de Tarapacá, Casilla 7-D, Arica 1000000, Chile; cflores@uta.cl

**Keywords:** proto-cells, morphogens, planets and exo-planets

## Abstract

This contribution considers proto-cell structures associated with asymmetries, mainly gravity, in the framework of reaction–diffusion. There are equivalent solutions for defined morphogen parameters in the equations that allow for defining proto-tissue complexity and configurational entropy. Using RNA data, improvements to the complexity and entropy due to the Earth’s gravity are presented. The theoretical proto-tissues complexity estimation, as a function of arbitrary surface gravity, is likewise proposed. In this sense, hypothetical aggregates of proto-cells on Mars would have a lower complexity than on Earth, which is equally valid for the Moon. Massive planets, or exoplanets like BD+20594b, could have major proto-tissue complexity and, eventually, rich biodiversity.

## 1. Introduction

Life on Earth began about one billion years ago [1,2], but what life is seems still to be an intricate question [3,4]. However, the understanding of the concept of life [4] as a self-sufficient physicochemical system, developed under Darwinian evolution theory, remains an adequate framework. This paper considers a way to achieve elementary prebiotic structures from an out-of-equilibrium point of view [5,6] when asymmetries, such as gravity, are present. In addition, amino acids exist in meteorites [7,8]; consequently, this work ignores a particular type of reactive element at this stage. I emphasize the generation, not of an isolated cell, but of a structure comprising elementary proto-cells. This is the framework of so-called complex systems, and the concept of morphogenesis [9,10,11,12]. Meanwhile, the relevant conception of inheritance will be explicitly considered in the sense of viability through adaptation in open systems.

Morphogenesis is related to the processes that turn undifferentiated cells into differentiated ones. Remarkably, cells do not contain the differentiation information which is stored in their relative and surrounding position [9,10,11]. That is, cell differentiation is a response to spatial location. Hence, privileging one direction, gravity must play a role in morphogenesis.

The information received by cells in the morphogenesis process is provided by basic physicochemical configurations. These spatial and temporal configurations are based on reagents called morphogens [9,10,11]. In practical terms, morphogen configurations are the previous scheme where the differentiation of cells is accommodated.

The following concepts [11] are adopted as a framework and used in the construction of a proto-tissue model in a finite spatial region [12]:

- Replication. Reactants interact to produce compound A mainly from another chemical compound, substrate B. Metabolism is assumed implicitly in the replication process. This process retains a sense of order which will be measured in terms of configuration entropy.
- Variation. Spatial patterns (containing “bricks” of A) have multiple and equivalent forms for fixed values of physical and chemical parameters. This is the analog to the concept of degeneration of states in physics.
- Heredity. On a large scale of perturbations, there is a viable continuity of equivalent traits (patterns) promoting adaptation.

Points (a) and (b) are linked to physicochemical procedures when compound A corresponds to the morphogen. Statement (c) is a necessary condition of living systems [11]. In this sense, inheritance is related to the formation of equivalent structures and viability. Here, an observation is necessary. Living systems, not their origins, can be characterized by the operative concept called autopoiesis [13,14,15,16,17,18], i.e., the robustness of internal cycles faces external disturbances, among other fundamental properties. From this point on, nonlinear reaction equations can provide limit cycles that satisfy this requirement.

In this paper, reaction–diffusion equations for chemical reagents are proposed. Consider two chemical compounds, A (the morphogen) and B (the substrate), in a given spatial region and the reaction process:

$$A\;\overset{k_{A}}{\to }\;\alpha \;\;\;\;\;B\;\overset{k_{B}}{\to }\;\beta ,\;A+2B\;\overset{k_{AB}}{\to }\;3A.$$

These reactions are somewhat different from Schnackenberg’s usual model [19,20,21,22,23], where the latter term operates as $2A+B\overset{k_{AB}}{\to }\;3A$. In the above process, the final configuration for “bricks” A, the morphogen, is built more quickly as it requires only one element A to generate the reaction.

In order to facilitate the understanding of this work, a brief description of the sections and their articulations will be given here. In Section 2, based on the reaction process between the chemical reagents, the evolution equations for the concentrations of morphogens and substrates are proposed. These equations incorporate spatial (diffusion) and nonlinear elements. Emphasis is placed on directional asymmetries and spatial growth conditions for the morphogen. Section 3 considers stationary solutions related to the degeneration ellipsoid that allows the researcher to define the number of equivalent states. Section 4 constructs an explicit example of proto-tissues based on the solutions in the previous section. Section 5 is the largest contribution in the field defined as “life”. The concept of complexity of a proto-tissue is defined simply, and intuitively, as that related to the number of accessible states of the system. In addition, the entropy is evaluated in direct terms of the parameters. In this section, estimates are made based on terrestrial parameters and RNA. In Section 6, the most hypothetical, the complexity of proto-tissues is evaluated based on the acceleration of gravity for different planets and exoplanets. Some conjectures are considered in relation to biodiversity and gravity. The last section is devoted to the relevant conclusions of this work.

## 2. Reaction–Diffusion for Proto-Tissues

The law of mass action, associated with the above-mentioned chemical processes, suggests the evolution equations for concentration variations A and B:

(1) $$\frac{\partial }{\partial t}A=\left(\left(D_{A}\nabla \right)\cdot \nabla -k_{A}\right)A+2k_{AB}A{\left|B\right|}^{2},$$

(2) $$\frac{\partial }{\partial t}B=\left(\left(D_{B}\nabla \right)\cdot \nabla -k_{B}\right)B-2k_{AB}A{\left|B\right|}^{2}.$$

Parameters $k_{A}$, $k_{B}$ and $k_{AB}$ are the usual chemical rates, and D is the diffusion tensors that promote cross-diffusion and spatial asymmetries such as gravity. The above set supports uniform solutions, i.e., A = constant and B = constant. In addition, and by simplicity, the concentrations are assumed to be dimensionless, for example, the number of molecules per mole.

The interest is in asymmetric spatial configurations where gravity plays a role in cell differentiation. For example, consider tiny asymmetric diffusion coefficients like

(3) $$D_{A}=\left(\begin{array}{cc} D_{A} & 0\\ 0 & D_{A}+\Delta D_{A} \end{array}\right),\;\;\;D_{B}=\left(\begin{array}{cc} D_{B} & 0\\ 0 & D_{B} \end{array}\right)$$

A key point exploited in this work is that through fixed solution $\left|B_{o}\right|$, Equation (1) is formally linear in *A* and supports superposition. That is, if there are multiple equivalent solutions A for a given $\left|B_{o}\right|$, these solutions can be added together due to the particular linearity of Equation (1). In addition, from the expected growth of species A; condition [12]

(4) $$2k_{AB}{\left|B_{o}\right|}^{2}>k_{A}$$

applies, which is required for new emerging structures due to the instability of old configurations. This means the growth rate for A is greater than its destruction rate. Furthermore, from Equations (1) and (2) results the constraint

(5) $$\frac{\partial }{\partial t}A+\frac{\partial }{\partial t}B=\left(\left(D_{A}\nabla \right)\cdot \nabla -k_{A}\right)A+\left(\left(D_{B}\nabla \right)\cdot \nabla -k_{B}\right)B$$

Finally, semi-analytical solutions of the Schnakenberg equation were considered in reference to Noufaey [23]. It contains limit cycles, an important notion for autopoiesis [13,14,15,16,17,18] as a framework to characterize life.

## 3. Stationary States: Equivalent Solutions and a Comprehensive Complexity Measure

General solutions of Equations (1) and (2) are difficult to achieve, so if the focus remains on stationary states with wave-vector $\vec{k}$, consider:

(6) $$A\left(\vec{x}\right)=A_{o}e^{i\left(\vec{k}\cdot \vec{x}\right)},$$

(7) $$B\left(\vec{x}\right)=B_{o}e^{i\left(\vec{k}\cdot \vec{x}\right)}.$$

From Equations (1) and (2) emerge the algebraic equations for amplitudes $A_{o}$ and $B_{o}$ given by

(8) $$0=\left[2k_{AB}{\left|B_{o}\right|}^{2}-\left(\left(D_{A}\vec{k}\right)\cdot \vec{k}+k_{A}\right)\right]A_{o},$$

(9) $$0=2k_{AB}A_{o}{\left|B_{o}\right|}^{2}+\left(\left(D_{B}\vec{k}\right)\cdot \vec{k}+k_{B}\right)B_{o}.$$

**Figure 1:**
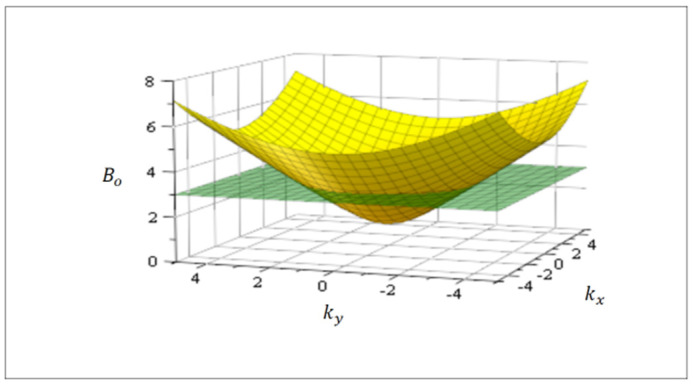
Amplitude $B_{o}\left(k_{x},k_{y}\right)$ in Equation (10) for an isotropic diffusion tensor $D_{A}$ in dimension two. For a fixed value of $B_{o}$, there is a sub-space (circle, green cutting plane) in the k-space displaying multiple equivalent solutions (complexity). These equivalent solutions can be superposed in Equation (1) which becomes lineal in this sub-space (Section 4).

The first equation, with assumed amplitude $\left|B_{o}\right|$, defines an n-dimensional ellipsoid in $\vec{k}$-space (see Equation (4)):

(10) $$\left(D_{A}\vec{k}\right)\cdot \vec{k}=\left(2k_{AB}{\left|B_{o}\right|}^{2}-k_{A}\right),$$

where consequently, there are many equivalent wave-vectors $\vec{k}$ for a given value of amplitude $\left|B_{o}\right|$. As an example, solving for $B_{o}$ in Equation (10), Figure 1 shows the function $B_{o}\left(k_{x},k_{y}\right)$ in spatial dimension two for an isotropic system where the equivalent states (degeneration) are defined by a circle related to a cutting plane. Note the formal similarity of Equation (10) with the Dirac equation for massive fermions, with $\sqrt{k_{A}}$ playing the role of mass.

On the other hand, for different values of $\vec{k}$, the morphogen amplitude $\left|A_{o}\right|$ formally follows from Equation (9) such as

(11) $$\left|A_{o}\right|=\frac{1}{2k_{AB}\left|B_{o}\right|}\left|\left(D_{B}\vec{k}\right)\cdot \vec{k}+k_{B}\right|,$$

marking the “uncertainty principle” $\left|A_{o}\right|\left|B_{o}\right|\geq k_{B}/2k_{AB},$ which requires $k_{B}\neq 0$ and, the equality becomes valid at low diffusion ($D_{B}\;\sim \;0$).

## 4. Constructing Proto-Cell Aggregates (Tissues)

For a given amplitude $\left|B_{o}\right|$ in the space of wave-vectors of spatial dimension d, there is a set of equivalent wave-vectors on the surface of the n-dimensional ellipsoid Equation (10). For instance, for a diagonal matrix $D_{A}$ like Equation (3), the following four fixed wave-vectors have equivalents in dimension two:

(12) $$\left(k_{o},p_{o}\right);\left(k_{o},-p_{o}\right);\left(-k_{o},p_{o}\right)\;\mathrm{and}\;\left(-k_{o},-p_{o}\right).$$

At this stage, for the same $\left|B_{o}\right|$ related to the above-defined four vectors, Equation (1) is lineal for concentration A. The superposed solution $e^{ik_{o}x+ip_{o}y}-e^{ik_{o}x-ip_{o}y}-e^{-ik_{o}x+ip_{o}y}+e^{-ik_{o}x-ip_{o}y}$ exists and can be re-arranged as:

(13) $$\left|A\right|=\left|A_{o}\sin\left(k_{o}x\right)\sin\left(p_{o}y\right)\right|,$$

**Figure 2:**
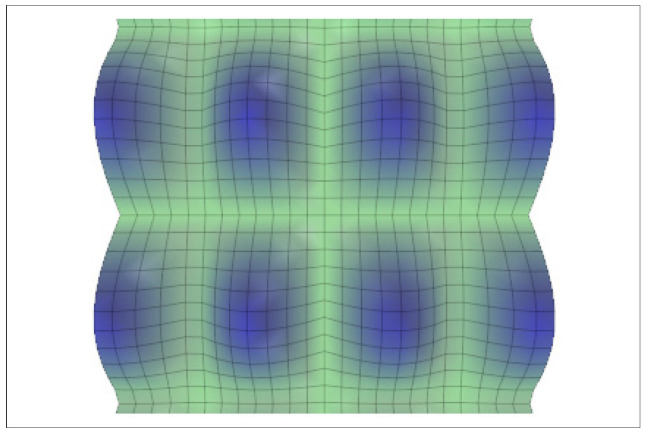
Proto-cell aggregates. If there is a set of equivalent solutions defined by Equation (10), the superposition of two or more of them is possible (Equation (1) becomes linear). The figure exhibits the morphogen superposition for $\left|A\right|:$ $\left|\sin\left(k_{o}x\right)\sin\left(p_{o}y\right)\right|$ (with $k_{o}=1;\;p_{o}=0.5$). It describes a proto-tissue configuration or pattern. Other configurations related to hexagons, for example, are also possible.

Figure 2 shows this kind of proto-tissue solution.

Finally, general stationary solutions can be written as $A=A_{o}\sum_{k\in ⊙}e^{i\left(\vec{k}\cdot \vec{x}\right)}$, with $\vec{k}$ in the n-dimensional ellipsoid defined by Equation (10). Remarkably, these kind of solutions are not approximates.

## 5. Proto-Tissue Complexity and Entropy from RNA Estimation on Earth

The condition for the generation of new structures or tissues was considered in detail by Flores [12], but here it is assumed the condition established in Equation (4) suffices.

Equation (10) defines an n-dimensional ellipsoid for the wave vector in the space of dimension d with volume: $\left(\pi \pi^{d/2}/\Gamma (d/2+1\right)\prod_{1}^{d}a_{i}$ ($a_{i}$ semi-axes). Assuming asymmetry in the z-direction ($\Delta D_{A}\neq 0$), the dimensionless volume C in phase space corresponds to:

(14) $$C\propto {\left[\frac{1}{D_{A}}\left(2k_{AB}{\left|B_{o}\right|}^{2}-k_{A}\right)\right]}^{\frac{d}{2}}\frac{1}{\sqrt{1+\Delta D_{A}/D_{A}}},$$

being, as usual in statistical mechanics [24,25,26,27], the number of equivalent configurations (complexity) for a given substrate $B_{o}$. Additionally, it allows defining the configuration entropy associated with these proto-cell structures. Note that in Equation (14), the spatial dimension d can also be assumed to be fractional [12].

At this point, a consideration must be made. $\vec{k\;}$-vectors run along the surface of the ellipsoid defined by Equation (10) and this surface is linked to entropy (Boltzmann’s entropy). However, in this paper we consider rather the volume of the ellipsoid (Gibbs’ entropy). In this sense, the measure related to Equation (14) corresponds to a finite range of $B_{o}$ values.

Without asymmetries $C=C_{0}$ (i.e., when $\Delta D_{A}=0$). The complexity parameter C Equation (14) is reformulated as

(15) $$C=\left(\frac{1}{\sqrt{1+\Delta D_{A}/D_{A}}}\right)C_{0}\;$$

**Figure 3:**
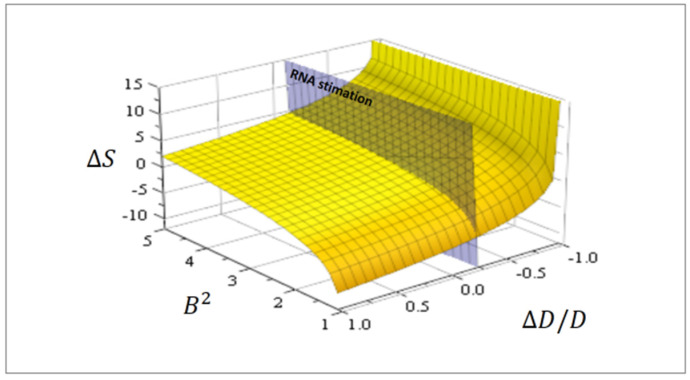
The effective proto-tissue configuration entropy $\Delta S=\left(S-S_{o}\right)$ Equation (16) as a function of asymmetry $\Delta D_{A}/D_{A}$ of the diffusion coefficient and normalized substrate concentration $B^{2}=\left(2k_{AB}/D_{A}k_{A}\right){\left|B_{o}\right|}^{2}$. The plane cutting the surface corresponds to $\Delta D_{A}/D_{A}\;\sim \;-0.25$, an RNA estimation related to Earth gravity.

Consequently, asymmetries change complexity or the number of equivalent configurations. Moreover, always from Equation (14), the configuration entropy $S=ln\left(C\right)$ becomes

(16) $$S=\frac{d}{2}ln\left(\frac{1}{D_{A}}\left(2k_{AB}{\left|B_{o}\right|}^{2}-k_{A}\right)\right)-\frac{1}{2}ln\left(1+\frac{\Delta D_{A}}{D_{A}}\right)+S_{o}\left(d\right)$$

with $S_{o}\left(d\right)$ being the residual entropy (Figure 3).

The Einstein relation between diffusion coefficient D and collision time τ is given by D=mkT/τ (*m* mass). Accordingly, for enlarged collision time Δτ>0, the diffusion coefficient diminishes ΔD<0 and the entropy grows ΔS>0 (Figure 3). In other words, from Equation (15), asymmetries promote complexities.

Explicitly, due to gravity g, assume

(17) $$\Delta D_{A}=-\sqrt{g}L^{3/2}$$

When L a characteristic linear size and an estimation for RNA $L\;\sim \;4\times 10^{-8}$ [m], then $\Delta D_{A}\;\sim \;-2.5\times 10^{-11}$ [m^2^/s]. A diffusion coefficient $D_{A}\;\sim \;10^{-10}$ [m^2^/s] seems always appropriate [28] for RNA, and then due to gravity $\Delta D_{A}/D_{A}\;\sim \;-0.25$. The plane cutting the surface in Figure 3 corresponds to this value. In addition, using Equation (15), the relative number of configurations attributable to gravity can be estimated in this case as $C/C_{o}\;\sim \;$1.15, hence gravity promoted complexity in proto-tissue constructions on Earth.

## 6. Proto-Tissue Complexity and Planetary Gravity: Comparison with Earth

Provided that Equations (15) and (17) hold, and expanding in the parameter g, complexity variations for proto-tissues become related to gravity through $\Delta C\propto \sqrt{g}$. This way, there is a simple correlation for complexity for two different planets (a and b) where proto-tissues could eventually be generated:

(18) $$\;\frac{\Delta C_{a}}{\Delta C_{b}}=\sqrt{\frac{g_{a}}{g_{b}}}.$$

**Figure 4:**
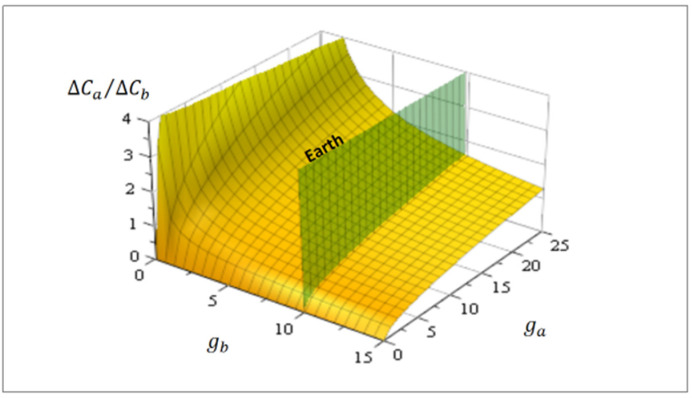
Eventual proto-tissues correlation for complexity $\Delta C_{a}/\Delta C_{b}$ for two hypothetical planets (Equation (18)) as a function of their surface gravity $g_{a}$ and $g_{b}$. The green cutting plane represents Earth ~9.8 [m/s^2^] and planets with relatively larger gravity have, in due course, proto-tissues with major structural complexity. In the Mars case, $g_{Ma}\;\sim \;3.7$ [m/s^2^], hypothetical proto-tissues have minor complexity compared to Earth because $\Delta C_{Ma}/\Delta C_{E}\;\sim \;0.61$.

Figure 4 shows this correlation as a function of arbitrary gravities $g_{a}$ and $g_{b}$. As an eventual estimation, on Mars $g_{Ma}\;\sim \;3.7$ [m/s^2^] and on Earth $g_{E}\;\sim \;9.8$ [m/s^2^]. Consequently, the correlation between degrees of proto-tissue complexity on both planets is hypothetically like $\Delta C_{Ma}/\Delta C_{E}\;\sim \;0.61$.

Always in Figure 4, the green plane cutting the surface corresponds to Earth’s gravity, so more massive planets or exoplanets [29,30] ($g_{a}>9.8$ [m/s^2^]) possess hypothetically more complex proto-tissue structures and, it may be surmised, supposedly major biodiversity.

Using Earth as patron of proto-tissue complexity with value $\Delta C_{E}=1$, Equation (18) can be reformulated for any body with surface gravity g [m/s^2^] as

(19) $$\Delta C=0.32\sqrt{g}$$

**Figure 5:**
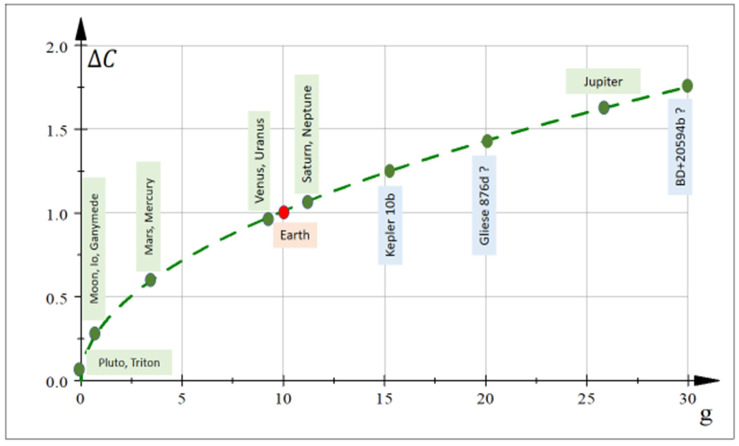
Hypothetical proto-tissue complexity ΔC (Equation (19)) for different planets, moons and exoplanets as a function of their respective gravitational parameter g [m/s^2^]. Earth is assumed to be a reference point, with proto-tissue complexity equal to 1 (red point). The Moon has relative complexity ΔC ~ 0.41 and Mars ΔC ~ 0.61. In these two corps, hypothetical proto-tissue configurations could be less complex than on Earth. Jupiter with large gravitational constant g ~ 26 [m/s^2^] could eventually possess a complexity of proto-cell aggregates ΔC ~ 1.63 larger than Earth.

Largely hypothetical, Figure 5 shows the supposed proto-tissue complexity as a function of (approximate) surface gravity for different astronomical bodies in the solar system and some exoplanets. For instance, Pluto has relative complexity ΔC ~ 0.25 and Triton ΔC ~ 0.28 and, theoretically, proto-tissues could be less complex there than on Earth.

## 7. Conclusions and Scopes

Generally, and keeping Earth as reference, there are optimal parameters for hypothetical life on planets or exoplanets [31,32,33,34,35,36,37]. In this way, this work contribution is connected to stationary proto-cellular structures with asymmetries (gravity [38]).

From the proposed reaction–diffusion equations, even featuring nonlinear equations, this model supports the superposition of solutions when the wave vector remains in a well-defined n-dimensional ellipsoidal sub-space (Section 3). In this sub-space exists equivalent solutions which allow for defining complexity and entropy (Section 5). This idea discussed in this paper has a degree of similarity to the notion of size-complexity discussed by Bonner [39]. Also, it is worth mentioning that there are other ways to quantitatively characterize the complexity in different systems [40]. Accordingly, using RNA-related data, corrections in the diffusion coefficient, complexity, and entropy due to Earth’s gravity were evaluated.

The results obtained in this work are valid for finite and relatively quiet environments where diffusion plays a fundamental role. On Earth, this may be important considering that conditions in the prebiotic era were probably extremely aggressive and variable. Phenomena such as turbulence can alter the nature of the solutions proposed here and eliminate the characteristic patterns obtained for proto-tissues.

Theoretic estimates of proto-tissue complexity Equation (19) was proposed as a function of surface gravity for arbitrary planets that allow comparisons with terrestrial proto-tissue (Section 6). In this sense, hypothetical proto-cell aggregates on Mars would have lower quantitative complexity than those on Earth, which would be equally valid for the Moon. On massive planets, or exoplanets such as BD+20594b, cellular proto-tissues would supposedly have greater complexity (Section 6, Figure 5).

Reaction–diffusion equations such as Equations (1) and (2) deserve an in-depth study from the dynamic point of view. That is, the formation of proto-tissue and its temporal evolution becomes the next step in this research. The notion of autopoiesis, particularly the idea of limit cycles acting in the context of life, should also be incorporated as a function of gravity.

Finally, and hypothetically, if great complexity of proto-tissues existed on (exo) planets with pronounced gravity, it is plausible to expect they also host large biodiversity when assuming the validity of Darwinian evolution theory in this context.

## Data Availability

Not applicable.
